# Two putative-aquaporin genes are differentially expressed during arbuscular mycorrhizal symbiosis in *Lotus japonicus*

**DOI:** 10.1186/1471-2229-12-186

**Published:** 2012-10-09

**Authors:** Marco Giovannetti, Raffaella Balestrini, Veronica Volpe, Mike Guether, Daniel Straub, Alex Costa, Uwe Ludewig, Paola Bonfante

**Affiliations:** 1 Department of Life Sciences and Systems Biology, University of Torino and IPP-CNR, Viale Mattioli 25, Torino, 10125, Italy; 2 Institute of Crop Science, University of Hohenheim, Fruwirthstrasse 20, Stuttgart, 70599, Germany; 3 Department of Life Sciences, University of Milano, Via Celoria 26, Milano, 20133, Italy; 4 Botanical Institute, Karlsruhe Institute of Technology, Hertzstrasse 16, Karlsruhe, D-76187, Germany

**Keywords:** Arbusculated cells, Legumes, Aquaporin, Symbiosis, XIP, NIP, Mycorrhizal fungi, *Lotus japonicus*

## Abstract

**Background:**

Arbuscular mycorrhizas (AM) are widespread symbioses that provide great advantages to the plant, improving its nutritional status and allowing the fungus to complete its life cycle. Nevertheless, molecular mechanisms that lead to the development of AM symbiosis are not yet fully deciphered. Here, we have focused on two putative aquaporin genes, *LjNIP1* and *LjXIP1*, which resulted to be upregulated in a transcriptomic analysis performed on mycorrhizal roots of *Lotus japonicus*.

**Results:**

A phylogenetic analysis has shown that the two putative aquaporins belong to different functional families: NIPs and XIPs. Transcriptomic experiments have shown the independence of their expression from their nutritional status but also a close correlation with mycorrhizal and rhizobial interaction. Further transcript quantification has revealed a good correlation between the expression of one of them, *LjNIP1*, and *LjPT4*, the phosphate transporter which is considered a marker gene for mycorrhizal functionality. By using laser microdissection, we have demonstrated that one of the two genes, *LjNIP1*, is expressed exclusively in arbuscule-containing cells. LjNIP1, in agreement with its putative role as an aquaporin, is capable of transferring water when expressed in yeast protoplasts. Confocal analysis have demonstrated that eGFP-LjNIP1, under its endogenous promoter, accumulates in the inner membrane system of arbusculated cells.

**Conclusions:**

Overall, the results have shown different functionality and expression specificity of two mycorrhiza-inducible aquaporins in *L. japonicus*. One of them, LjNIP1 can be considered a novel molecular marker of mycorrhizal status at different developmental stages of the arbuscule. At the same time, *LjXIP1* results to be the first XIP family aquaporin to be transcriptionally regulated during symbiosis.

## Background

Knowledge has increased concerning the fact that a plant does not act as an individual on its own, but as an actor in a vast stage populated by bacteria, fungi and other microorganisms [1-3]. Arbuscular mycorrhizal (AM) fungi represent one of the most important components of the complex root-plant microbiome, since they are present in about 80% of vascular plants. They supply the plant with phosphate, nitrogen, mineral salts and water, and they guarantee a more extensive protection from biotic and abiotic stresses at both local and systemic level. On the other hand, the plant allows the fungus to access the photosynthetic carbon-compounds [4].

A partly known chemical dialogue guides this close relationship: plant strigolactones trigger the growth and branching of spore-germinating mycelium, while fungal signals, including lipochitooligosaccharides, stimulate root growth and branching [5]. The latter are recognized by a yet unknown receptor that causes a well conserved signal cascade and induces Ca^2+^ oscillations within the nuclei, in a similar way to the mechanisms involved in rhizobium-legume symbiosis [6]. Once they have penetrated the epidermis, AM hyphae rapidly develop into plant cortical cells and form a tree-shape structure called arbuscule, which is the functional site of nutrient exchange. Its formation is a non-synchronous process and its life-span is assumed to last no more than 10 days [7]. The arbuscule accommodation process requires a substantial remodelling of the cortical cell: all the thin arbuscule branches are enveloped by a periarbuscular membrane (PAM), which does not simply surround the arbuscule as a whole, but closely follows the surface of each branch, moulding to the arbuscule itself. PAM development marks the appearance of the symbiotic interface, the narrow intracellular compartment that allows AM fungi to grow inside the plant cell without breaking its integrity. Cell biology investigations have recently demonstrated that PAM biogenesis requires the proliferation of the endoplasmic reticulum, Golgi apparatus, trans-Golgi network and secretory vesicles [8], while the insertion of specific PAM proteins, such as MtPT4, occurs thanks to polarized secretion processes [9].

All these dramatic events are pointed out by the high number of genes that are regulated, not only in the whole mycorrhizal roots (e.g. [10-13]), but more specifically within the arbusculated cells [14-16]. In this framework, Hogekamp and coworkers have shown that two of the most upregulated membrane transporter genes of *Medicago truncatula* cells hosting AM arbuscules are aquaporins. This specific gene regulation has also been confirmed by various other reports in different mycorrhizal plants [14,17-19].

Aquaporins (AQPs) are well known for their ability to transport water, as well as other small solutes (i.e., ammonia, urea, boron), across the membranes of various organisms, and genetic defects involving aquaporin genes have been associated with several human diseases [20]. Plant AQPs are present in various tissues and play a role not just in transport, but also in cell differentiation, cell enlargement, leaf function, nutrient transport and metal toxicity [21-23]. A new database is now available to make enquiries on possible functions through a comparison of sequences and structures [21].

AQPs are a family of small pore-forming integral membrane proteins. The molecular basis of their selectivity mainly depends on two filters within the pore: the first is formed by the conserved dual “NPA” filters (asparagine, proline and alanine residues), while the second is formed by a constriction region that is also called the ar/R (aromatic/arginine) filter [22]. It appears that the properties of the four residues that make up the ar/R selectivity filter control the substrate specificity of the pore [23], and are thought to be useful for predicting the function of the protein [24]. On the basis of sequence comparisons, the AQPs of dicots and monocots can be divided into five conserved subgroups, and some of these subgroups appear to be consistently linked to specific subcellular localizations, hence their names: plasma membrane intrinsic proteins (PIPs), tonoplast intrinsic proteins (TIPs), nodulin 26-like intrinsic proteins (NIPs), small and basic intrinsic proteins (SIPs) and X intrinsic proteins (XIPs) [25,26]. The overall level of NIP expression in plants is lower than the expression of other AQPs, as they are usually associated with specialized organs and cells, and are involved in the exchange of metabolites between the host and the bacterial symbiont [27]. Among the so far characterized NIPs, AtNIP2;1 specifically accumulates in the endoplasmic reticulum of roots, whereas AtNIP5;1 is a plasma membrane MIP mainly expressed in root elongation zones [28-30]. XIP proteins instead have only been partially characterized, but it seems that they may have various expression patterns and functional characteristics.

Although mycorrhizas have been demonstrated to be crucial for the hydraulic properties of plant roots, as they enhance the tolerance of the host plants to water deficit [31], the involvement of AQPs in AM symbiosis is still unclear and under debate [32]. It is not known whether the beneficial water status of AM plants is enhanced by the regulation of root aquaporins or because of an enhanced water flow.

With the final aim of shedding light on the potential role of this gene family which seems to be highly AM-responsive, we have focused our research on two AQPs that were found to be upregulated in *Lotus* mycorrhizal roots [14], and which belong to the NIP and XIP classes. By means of a combination of different experimental approaches (expression patterns over various nutrient and symbiotic status, transcript localization and quantification of microdissected cells, functional characterization with heterologous assays and GFP-protein localization at a subcellular level through confocal microscopy), we have demonstrated that the two AQPs are genetically and functionally diverse, although they are both AM-responsive. *LjXIP1* is exclusively overexpressed in mycorrhizal roots, while *LjNIP1* is also *Rhizobium* responsive. LjNIP1 is solely present in AM roots and, more precisely, in arbusculated cells, where the protein is associated with the complex endomembrane system. Unlike a phylogenetically similar protein [33], LjNIP1 is involved in water, but apparently not ammonia, transport. These results open new questions on the functional role of AM-responsive AQPs and their relationship with arbuscules as the main fungal colonization structures.

## Results

### Gene isolation and phylogenetic analysis of LjNIP1 and LjXIP1

**Figure 1:**
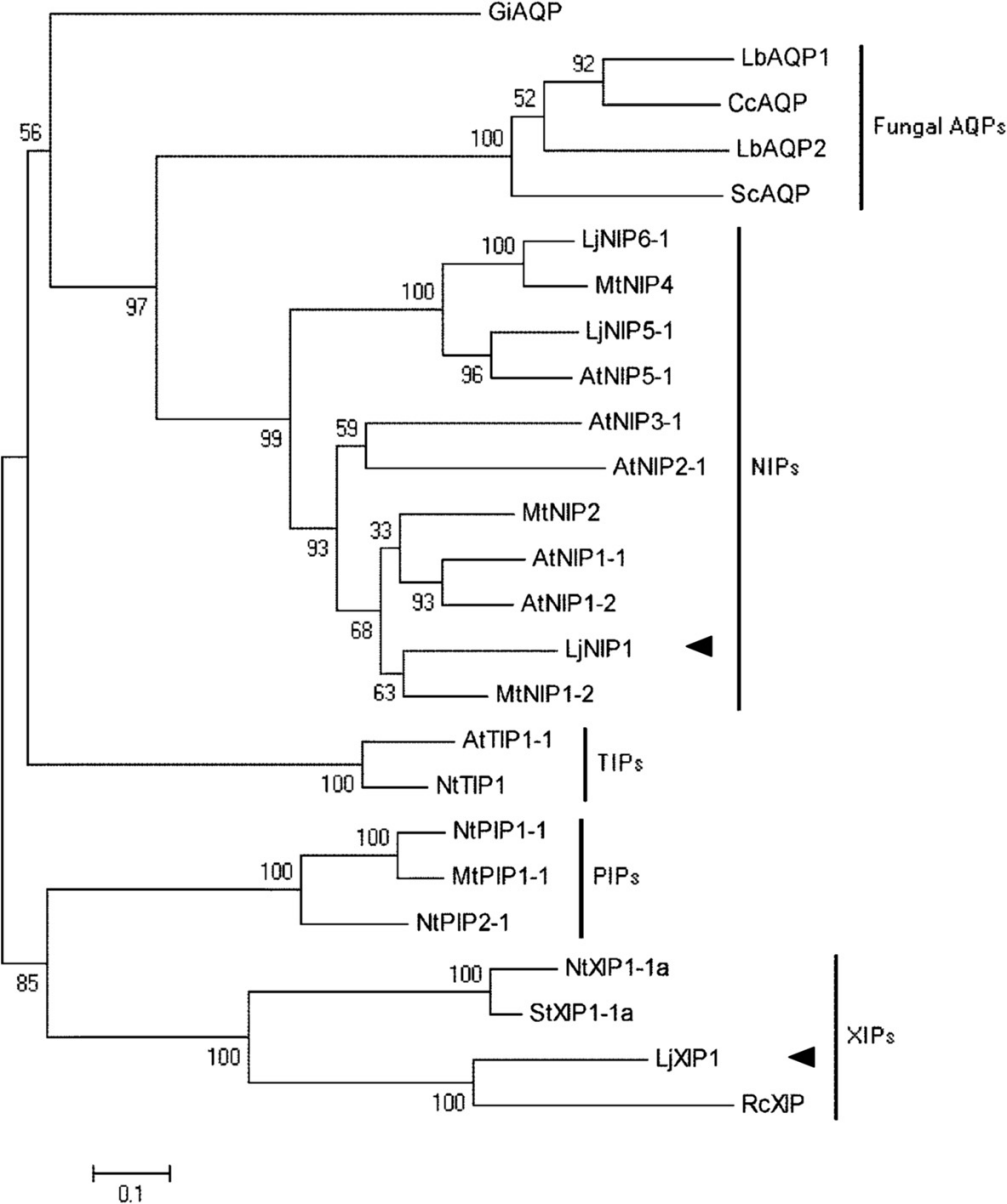
**Phylogenetic analysis of plant and symbiont fungal aquaporins.** An unrooted phylogenetic tree was generated for the aquaporin plant and fungal sequences with Mega 5,0 software using Muscle for the alignment and the neighbour-joining method for the construction of the phylogeny (http://www.megasoftware.net/). Bootstrap tests were performed using 1000 replicates. The black arrows mark the two proteins investigated in this study. Abbreviations for plant species: At, *Arabidopsis thaliana*; Lj, *Lotus japonicus*; Mt, *Medicago truncatula*; Nt, *Nicotiana tabacum*; Rc, *Ricinus communis;* St, *Solanum tuberosum;* and fungal species: Lb, *Laccaria bicolor*; Cc, *Coprinopsis cinerea*; Sc, *Schizophyllum commune*; Gi, *Glomus intraradices*. The GenBank accession numbers for the AQPs used are as follows: GiAQP [257219859]; LbAQP1 [170095169]; CcAQP [169862326]; LbAQP2 [170107189] ScAQP [302686158], LjNIP6-1 [162568625]; MtNIP4 [47531135]; NtXIP1-1a [309385599]; LjNIP5-1 [162568623]; AtNIP5-1 [126352290]; AtNIP3-1 [259016288]; AtNIP2-1 [32363364]; MtNIP2 [44887593]; AtNIP1-1 [32363362]; AtNIP1-2 [32363340]; MtNIP1-2 [355482834]; AtTIP1-1 [135860]; NtTIP1 [162809290]; NtPIP1-1 [17017255]; MtPIP1-1 [357492595]; NtPIP2-1 [17017257]; NtXIP1-1a [309385599]; StXIP1-1a [309385603]; RcXIP [255586851].

The 798-bp full-length cDNA of the *LjNIP1* gene (EMBL accession number HE860041) and the 900-bp full-length cDNA of the *LjXIP1* gene (EMBL accession number HE860042), obtained using a 5^′^ and 3^′^ RACE, encode respectively a 265 and a 299- amino-acid-long peptide. LjNIP1 has two conserved NPA filters, whereas LjXIP1 shows an atypical amino-terminal NPA (Additional file 1). If a transmembrane prediction software (http://bioinformatics.biol.uoa.gr/TMRPres2D/) is used, both aquaporin proteins are likely to have 6 full transmembrane domains, thus confirming the standard structure of this family (Additional file 2). The phylogenetic analysis (Figure 1) grouped them into two different functional sub-families. The highest homology of LjNIP1 was found with MtNIP1-2, previously called MtNIP1, which has already been partially described by Uehlein *et al.*[33] to be involved in ammonia transfer, while LjXIP1 showed significant similarities with different proteins belonging to the XIP family, a recently discovered family of unknown functions [25,34].

### AM symbiosis and the cell pattern of LjNIP1 and LjXIP1 expression

In a previous microarray study [14], the sequences identified as *LjNIP1* and *LjXIP1* resulted to be among the most upregulated genes with log2 values of 3.80 and 3.08, respectively, upon mycorrhization. In order to validate the specificity of such up-regulations, their expression patterns were investigated by comparing them with those of nodulated roots and respective controls, with high and low N and P availability. Mycorrhizal and control roots were sampled at 28 days, this time being considered the point at which there were the most arbuscules in the microarray experiment [14]. Control and nodulated roots were sampled at 35 dpi. From 32 to 36 nodules were counted in each inoculated root.

**Figure 2:**
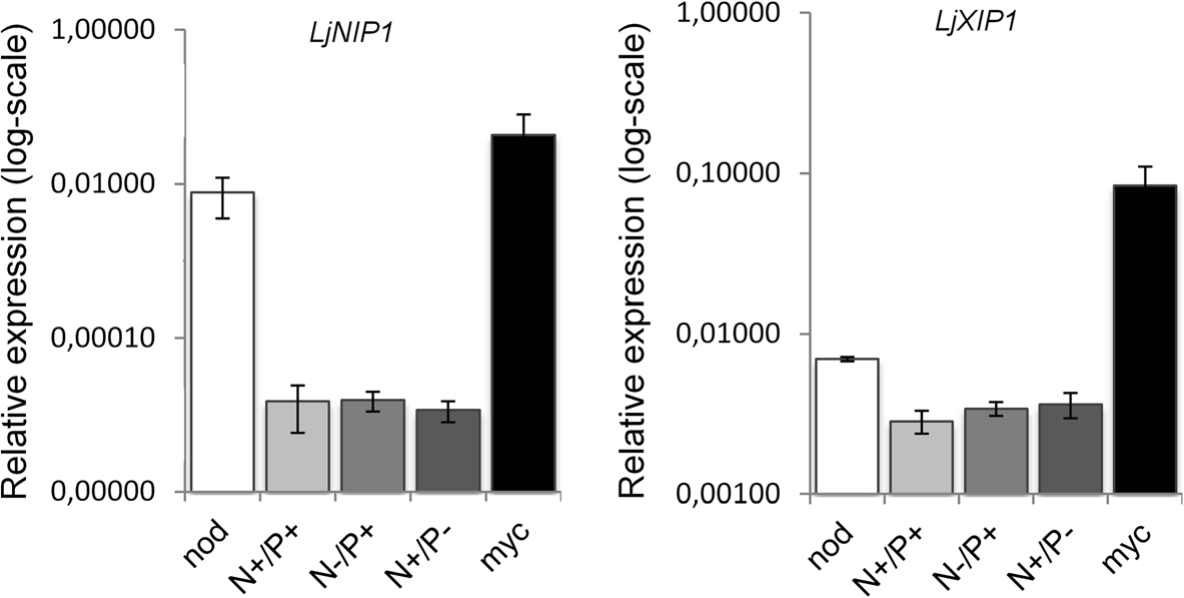
**Relative expression of *LjNIP1* and *LjXIP1.*** The relative expression of *LjNIP1* and *LjXIP1*, assessed by means of qRT-PCR, in *L. japonicus* after 28 days of mycorrhization and 35 days after nodulation. The c_t_ values of the samples were normalized against the c_t_ values of the housekeeping gene *UBQ10*. The data for each condition are presented as the mean and were obtained from three biological and three technical replicates. –N = 10 μM KNO_3_; +N = 4 mM KNO_3_; –P = 20 μM PO_4_^3-^; +P = 500 μM PO_4_^3-^ as indicated by Guether *et al.*[58]. Bars are representing the standard deviations.

Quantitative RT-PCR confirmed that the highest expression levels were reached in mycorrhizal roots. *LjNIP1* expression was also highly induced by nodulation (Figure 2), whereas *LjXIP1* expression was only slightly induced. Gene expression was not activated in the non-inoculated plants that were maintained at different N and P levels.

These results demonstrate that the expression of the two putative aquaporins depends on the presence of the root symbiont, but is probably not significantly influenced by an improved N and P status of either mycorrhizal or nodulated roots. In addition, the expression of *LjNIP1* and *LjXIP1* is related to the identity of the symbiotic microbe or the specific plant-microbe interaction.

### Cell-specific expression of *Lotus aquaporins*

**Figure 3:**
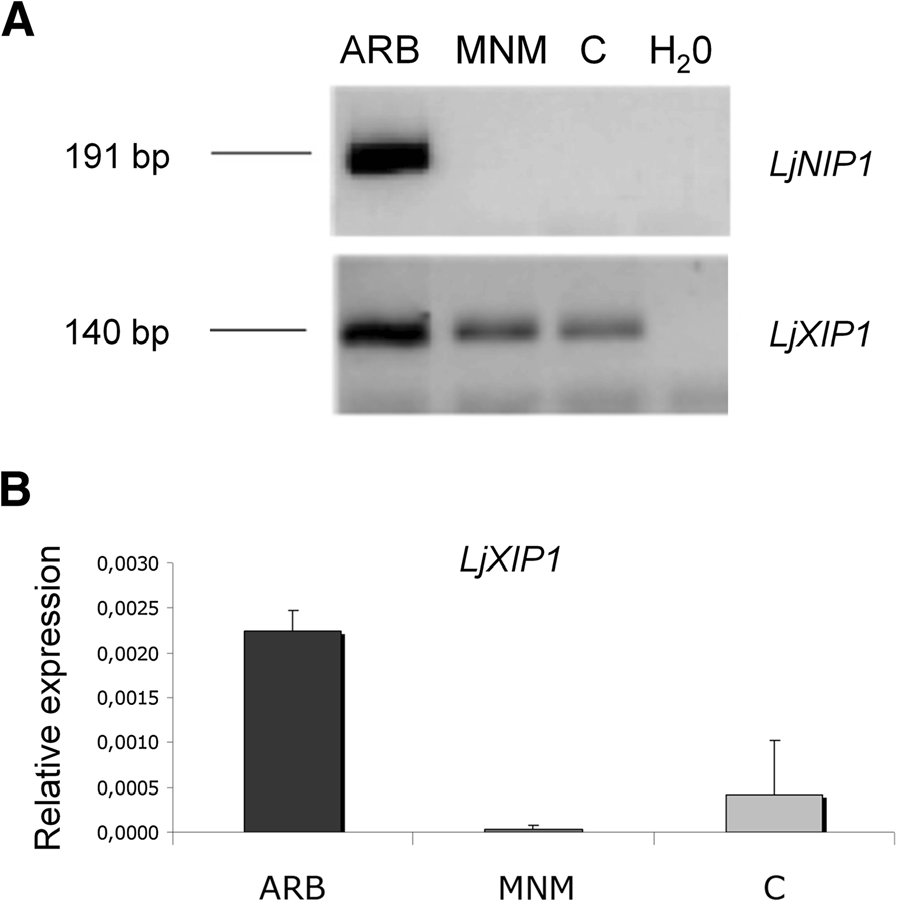
**RT-PCR and qRT-PCR on laser microdissected cells.** (**A**) RT-PCR analysis of *LjNIP1* and *LjXIP1* in LMD samples: *LjNIP1* amplified fragments were only detected in arbusculated cells (ARB), whereas *LjXIP1* fragments were also detected in non-colonized cortical cells from mycorrhizal roots (MNM) and in cortical cells from non-mycorrhizal roots (C). (**B**) Relative expression of *LjXIP1* in LMD samples assessed by means of One-Step qRT-PCR. The highest level was detected in the arbusculated cells. The values are the means of two biological samples and two technical replicates, with bars representing the standard deviations.

A laser microdissection approach was used to localize the transcript accumulation sites. Since the key structure of the nutrient exchange that takes place between plant and fungus has been demonstrated to be the arbuscule [4], cortical cells were chosen as a first target. Three cell types were collected by means of a laser microdissection system: arbusculated cells (ARB), non-colonized cortical cells from mycorrhizal roots (MNM), and cortical cells from non-mycorrhizal roots (C). When specific primers for *LjNIP1* and for *LjXIP1* were used, fragments of the expected size were present with different expression patterns: *LjNIP1* resulted to be specifically expressed in arbuscule-containing cells, whereas *LjXIP1* transcripts were present in all three cell types analyzed (Figure 3A). In order to better investigate this result, a further relative quantification of *LjXIP1* transcripts was performed through One-Step qRT-PCR on RNA extracted from microdissected cells (Figure 3B). This approach revealed that the gene was overexpressed in arbusculated cells in a significant way compared to the other cell types analyzed, thus confirming a possible additional or enhanced role within the arbuscule-containing cells. The validity of this result can be pointed out by the fact that RNA was not amplified before the quantification, and that the exact amount of RNA was preserved in each cell type.

### *LjNIP1* expression over colonization time

**Figure 4:**
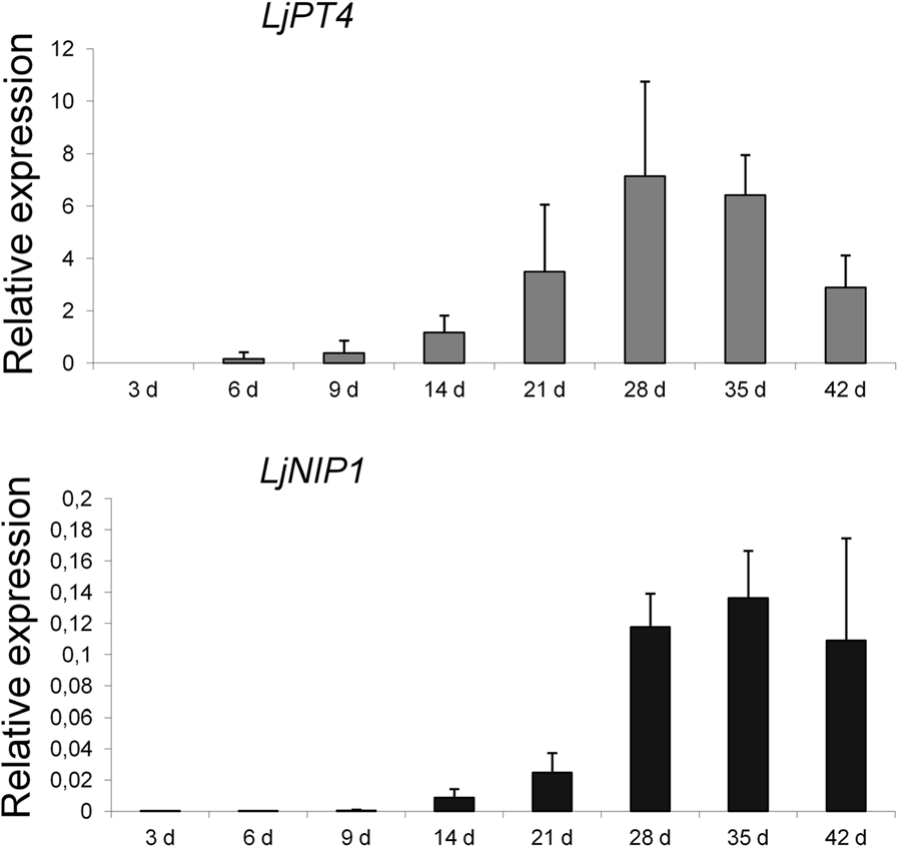
**Time course of *LjNIP1* and *LjPT4.*** Relative expression of *LjPT4* and *LjNIP1* over the colonization time assessed by means of qRT-PCR at different time points post inoculation. The Ct values of the samples were normalized, as explained in Figure 2. The values are the means of three biological replicates, with bars representing the standard deviation.

Since *LjNIP1* expression resulted to be more closely connected to the presence of the arbuscules, we followed the gene expression during a time course experiment. A comparison with the *LjPT4* expression levels was also performed to gather information on the relationship between *LjNIP1* expression and arbuscule development. *LjPT4* is the homologous of *MtPT4*, which is considered a marker of active arbusculated cells in *Medicago*, since its expression mirrors the arbuscule status, i.e. its expression decreases when the arbuscule collapses [35]. Figure 4 shows that the *LjNIP1* expression pattern mostly mimics *LjPT4* dynamics. The expression levels increase for both genes until 28 dpi, but interestingly, important differences were detectable in the expression at 35 and 42 days post inoculation: *LjPT4* expression started to decrease, probably due to the asynchronous process of the formation and collapse of the arbuscules, while *LjNIP1* expression did not show any significant difference between the two time points, and maintained high levels throughout the experiment. This allows us to speculate on the possibility of a role of this protein that is independent of the arbuscule status, being expressed both during arbuscule full functioning and senescence.

### Heterologous complementation of yeast mutants defective in ammonium or urea uptake

**Figure 5:**
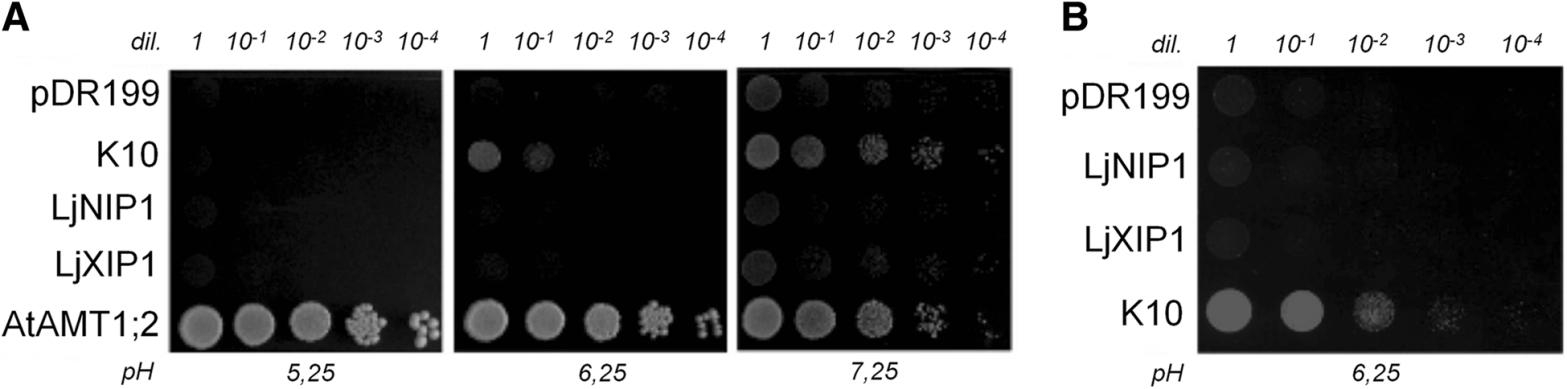
**Ammonia and urea permeability.** (**A**) Growth of ammonium uptake-deficient yeast (31019b; ΔΔΔmep1;2;3) transformed with AtAMT1;2, LjNIP1, LjXIP1, K10 and the empty control plasmid pDR199 on 1 mM ammonium as the only N source at different pH. (**B**) Growth of urea uptake-deficient yeast (YNVW1; Δdur3) transformed with LjNIP1, LjXIP1, K10 and the empty control plasmid pDR199 on 1 mM urea as the only N source. The ability of mutant yeasts to grow on the two different media was not restored by LjNIP1 or by LjXIP1. Serial dilutions (dil.) of cell suspensions, ranging from 1 to 1 × 10^-4^, are shown for both pictures.

Because of the high similarity of *LjNIP1* and *MtNIP1-2*[33], which is capable of transporting ammonium, we tested the capacities of both *Lotus* aquaporins to transport ammonium. Yeast (*Saccharomyces cerevisiae*) strain 31019b, which is defective in all three endogenous ammonium transporters (Mep1, Mep2, and Mep3), and is thus unable to grow on a medium containing <5 mM ammonium as the sole N source [33], was used. This strain was transformed with yeast vector pDR199, which expressed *LjNIP1* and *LjXIP1* under the control of the constitutive yeast PMA1 promoter. In order to compare the transformed yeasts with the already described plant ammonium transporters and ammonia channels, this yeast strain was also transformed with pDR199 expression vectors containing the following coding sequences: the NH_4_^+^ transporter *AtAMT1;2*[36] and *K10*, a mutant aquaporin capable of transporting ammonia, in addition to urea [37]. The mutant *K10* was derived from the water-selective *AtPIP2;1* and was mutated in the selectivity filter to have a typical ar/R region of AtNIP1s. Considering the acidic conditions of the periarbuscular space [38], yeast growth was analyzed on a yeast nitrogen base (YNB) medium under different pH conditions. As shown in Figure 5A, yeast growth was not restored by LjNIP1 or by LjXIP1 on the minimal medium containing 1 mM ammonium. As expected yeasts expressing AtAMT1;2 grew well at any pH and yeasts expressing K10 allowed a good complementation at pH 7,25. We performed the same experiment with yeast strain YNVW1, which is defective in its urea transporter and is thus unable to grow on a medium containing <5 mM urea as the sole N source [39]. As shown in Figure 5B, yeast growth was not restored by either *LjNIP1* or by *LjXIP1* on the minimal medium containing 1 mM urea as the sole N source but just by the positive control K10.

### LjNIP1 can transport water

**Figure 6:**
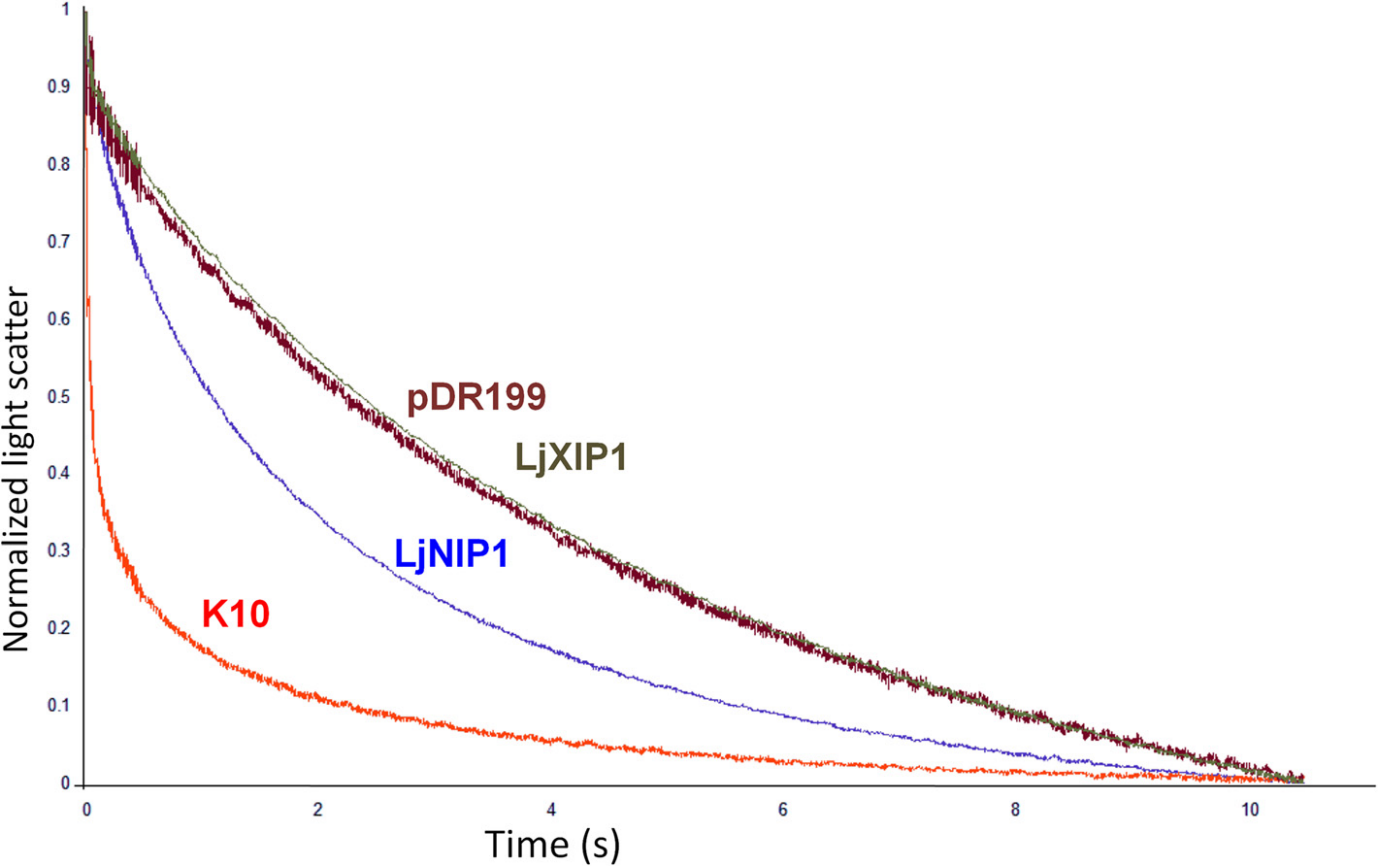
**Water permeability of LjNIP1 and LjXIP1.** Water permeability of intact yeast protoplasts expressing *LjNIP1* (blue), *LjXIP1* (dark green), *K10* (red) and the empty vector *pDR199* (maroon). Yeast swelling kinetics were recorded as time courses of the decreased scattered light intensity in a stopped flow spectrophotometer. The differences of scattered light are correlated with the capacity of protoplasts to acquire water in the rapid mixing device. LjNIP1 resulted to be able of transferring water whereas LjXIP1 did not show any difference with the empty vector pDR199.

In order to better characterize the *LjNIP1* and *LjXIP1* functions, the respective proteins were expressed in yeast protoplasts and the cells were examined, to assess their water transport capability, by means of a stopped-flow spectrophotometer [40]. This instrument allows measuring scattered light differences over time, which are correlated with the capacity of protoplasts to acquire water, in a rapid mixing device. The analysis of K10, as previously mentioned, served as an internal control. The experiment revealed that LjNIP1 increased the water permeability of the membrane, whereas the LjXIP1 aquaporin did not increase the water transport rates of the yeast cells subjected to hyperosmotic conditions (Figure 6).

These results lead to the conclusion that LjNIP1 may be involved in water transport, rather than in nitrogen solute transport. LjXIP1, however, was non-functional in all the assays. Whether this reflects poor localization into the plasma membrane, selectivity or a lack of an opening stimulus remains an open question.

### Subcellular localization of LjNIP1 in tobacco leaf epidermal cells

**Figure 7:**
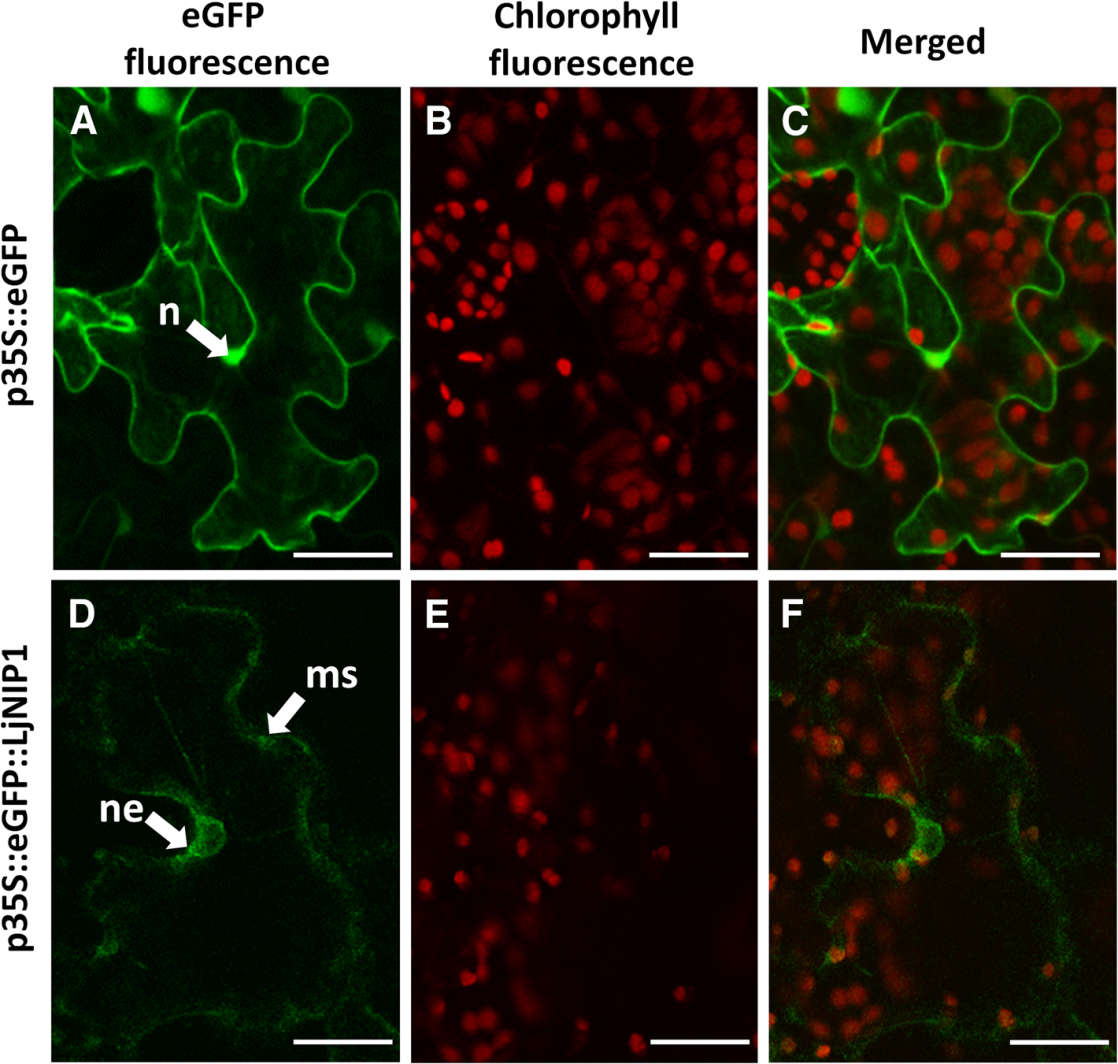
**Transient p35S-eGFP-LjNIP1 expression in *Nicotiana tabacum* epidermal leaf cells. A**-**C**. Free-eGFP fluorescence is homogenously present inside the epidermal cells and is accumulated above all in the plasma membrane and inside the nucleus (n), whereas the red chlorophyll autofluorescence is limited to the chloroplasts. **D**. A light green signal, due to eGFP-LjNIP1 expression, is accumulated in the inner membrane system (ms), starting from the nuclear envelop (ne). A weak signal is also associated to the plasma membrane. **E**. Chlorophyll fluorescence of the same leaf shown in D. **F**. Overlay image of D and E. Bars = 25 μm.

A chimeric *LjNIP1*-*eGFP* was constructed and placed downstream of the CaMV 35S promoter in order to gain insight into the subcellular localization of LjNIP1 in plant cells. Transient expression analyses were performed on epidermal cells from tobacco leaves. The fluorescence in the control cells, expressing free eGFP, was uniformly extended into the cell, including the whole nucleus and the cell wall (Figure 7A-7C). Figure 7D(-7F) shows tobacco cells leaves that express the LjNIP1-eGFP protein: a light fluorescence signal was found in the nuclear envelope surrounding the cell nucleus, a typical signal of the endoplasmic reticulum region, and the inner membrane system.

### eGFP-LjNIP1 accumulates in the endoplasmic reticulum and in the inner membrane system of *Lotus* mycorrhizal roots

**Figure 8:**
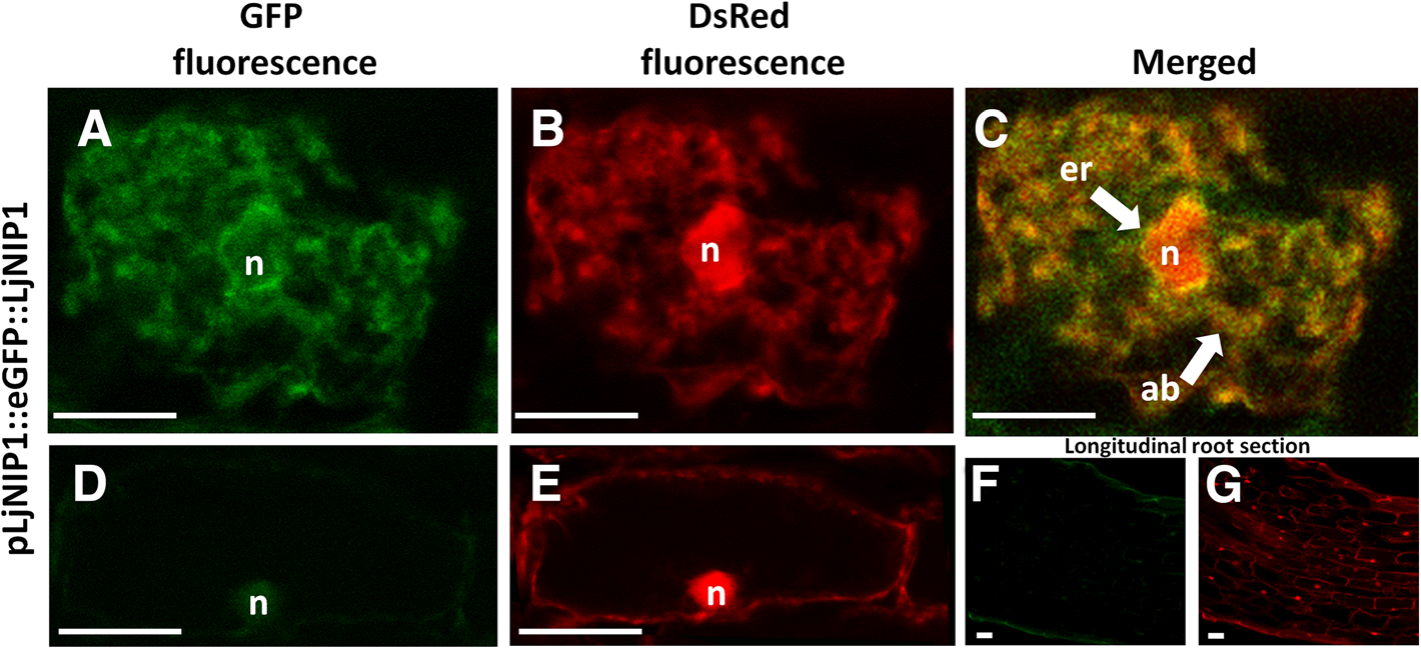
**Subcellular localization of LjNIP1 in mycorrhizal roots.** Confocal microscopy images showing *pLjNIP-eGFP-LjNIP1* expression in *L. japonicus* roots colonized with *Gi. margarita*. *pUBI10-DsRed* was expressed ubiquitously in the transformed roots and highlighted the cell nuclei (n). **A**-**C**: pLjNIP1-eGFP-LjNIP1 is present inside the whole cell lumen, being associated to the surface of the fungal arbuscule branches (ab) in a non-continuous pattern. It also accumulated outside the cell nucleus, resulting associated to the inner cell membrane system and to the endoplasmic reticulum (er). **D**-**E**: non colonized cortical cells from mycorrhizal plants; the nucleus (n) is in the typical peripheral position; the absence of *LjNIP1* expression, is revealed by the absence of any GFP signal, as shown also by the longitudinal section of a non colonized root where no GFP signal was detected (**F**) and DsRed was ubiquitously expressed (**G**). Bars = 25 μm.

In order to validate the intracellular localization of the LjNIP1 protein suggested by the aforementioned experiment on tobacco leaves, we generated *Lotus* transgenic roots that expressed the eGFP-LjNIP1 fusion protein under the control of the *LjNIP1* endogenous promoter. *Lotus* roots were transformed with *Agrobacterium rhizogenes* carrying the plasmid. The roots were inoculated with 10–20 *Gi. margarita* spores, and a minimum of ten independent transformed root lines were observed after 35–42 dpi. The transformed roots were identified through the expression of the DsRed protein under the control of the ubiquitin promoter. The GFP signal was only detected in cells harboring arbuscules (Figure 8A-8C), and the signal was evident in the membranes surrounding the nuclei, which are usually coincident with the endoplasmic reticulum, as well as in the inner cellular membranes, also likely including the vacuolar tonoplast. However, a possible signal from the periarbuscular membrane cannot be excluded, even if the fluorescent signal did not accurately follow all the arbuscule branches. No GFP fluorescence was observed in the epidermal and cortical tissues of non-colonized roots, as also confirmed at higher magnification where the nucleus occupies a peripheral position inside the cortical cells (Figure 8D,-8G).

## Discussion

When AM fungi colonize their host’s roots, a significant cell reorganization as well as important changes in the transcriptomic profiles are usually reported [41]. These cellular and molecular changes are particularly impressive in arbusculated cells, which are considered to be at the heart of the symbiosis. Since aquaporins - the water-channel proteins that allow the passage of water molecules through cell membranes - have often been reported to be upregulated in mycorrhizal roots, we characterized two *Lotus* genes, *LjXIP1* and *LjNIP1*, which resulted to be regulated during a microarray experiment [14]. The results demonstrate that the two genes encode aquaporins that are genetically and functionally diverse, even though both are overexpressed during AM symbiosis.

### Mycorrhiza-induced LjXIP1 and LjNIP1 belong to two different families of plant aquaporins containing different ar/R constriction regions

The name of the NIP protein family comes from the archetype nodulin26, which was initially characterized in soybean colonized by rhizobacteria [42]. The role of nodulin26 has been reported to be that of mediating the passage of water through the interface between the two symbionts. This group of integral membrane proteins is unique to plants and dates back to the first development phase of primitive land plants, suggesting a stable role during their evolution [24]. NIPs are widely distributed in both leguminous and non-leguminous plants, and 9 and 13 NIP genes are encoded by the *Arabidopsis* and rice genomes, respectively [43]. Among plant aquaporins, proteins belonging to this subfamily have nonpareil functional features [28]. The NIPs have been divided into three groups on the basis of their selectivity filter structure. *LjNIP1* belongs to class I, and shows great similarities with the homologous *Medicago* gene, which resulted to be overexpressed in mycorrhizal roots and is able to transfer ammonium or ammonia [33].

The function of XIP proteins, at a cellular level, has yet to be elucidated, but some of their features have been characterized: in 2011, Bienert and coworkers [34] showed that *Solanaceae* XIPs are able to transfer several uncharged solutes, such as urea, glycerol, and boric acid, but resulted to be non-permeable to water. They also speculated on a possible functional overlap between XIPs and NIPs because of their similarity in the substrate spectrum and in the selectivity pore. Conversely, XIPs from poplar resulted to have a wide and diverse expression pattern and to differ in their capability to transfer water [44]. LjXIP1 shows great similarity with an uncharacterized aquaporin from *Ricinus communis*.

The four aminoacids that form the ar/R constriction region are crucial for aquaporin substrate selectivity. As far as LjNIP1 is concerned, the selectivity filter is made up of tryptophan, isoleucine, alanine, and arginine. These residues form a wide, rather hydrophobic pore, and are typical of class I NIP proteins, which are characteristically able to transfer water [24], consistently with our functional assays (Figure 6). On the other hand, the LjXIP1’s ar/R selective pore is unusual, as it consists of a phenylanine, an arginine and two valines (an aromatic, a hydrophobic and two small residues) (Additional file 1), making it difficult to speculate on any possible compound affinities. However, it does show some similarities with typical NIP protein belonging to class III [43] and, intuitively, with other XIP proteins [34].

It has recently been reported that aquaporins from ectomycorrhizal fungi could play a role in the interaction with plants [45]. As expected, the aquaporins so far characterized in symbiotic fungi belong to a distant branch, compared to plant aquaporins (Figure 1), which demonstrated a different evolutionary story.

In conclusion sequence analysis clearly shows that LjNIP1 and LjXIP1 belong to two diverse groups of plant aquaporins and the aminoacidic composition of the constriction region predicts different substrates, as confirmed by our functional experiments (see below).

### Mycorrhiza-induced *LjNIP1* is specific of arbusculated cells and targets the inner membrane system

Since aquaporins have already been detected as being expressed during nodule symbiosis [46], we wanted to understand whether *LjXIP1* and *LjNIP1* were also *Rhizobium*-responsive and whether their expression depended on P and N availability. Neither the *LjXIP1* nor the *LjNIP1* expression levels resulted to be influenced by the soil nutrient concentration, but, interestingly, they were both overexpressed during interaction with microorganisms: *LjXIP1* resulted to be elicited above all by AM fungi, whereas *LjNIP1* was also regulated by rhizobial bacteria. This clearly shows that the expression of the two aquaporins is related to the identity of the symbiotic microbe. Transcript localization and relative quantification, through laser microdissection experiments in *L. japonicus* mycorrhizal roots, have shown that *LjXIP1* is expressed in different root cell-types, with higher expression levels in arbusculated cells but also constitutive levels in non-colonized cortical cells. *LjNIP1* is instead exclusively expressed in arbuscule-containing cells, which raises the question of whether such close cell-type dependence is maintained in bacteroid-containing cells.

Looking at experiments in which a laser microdissection approach was adopted [15,16], it can be concluded that the data pertaining to aquaporin expression patterns are extremely consistent, regardless of the plant-fungus association, suggesting that they could be considered good novel indicators of AM symbiosis and, in the case of *LjNIP1,* of root symbiosis.

Current knowledge indicates that AM-responsive phosphate transporters are excellent markers of AM functionality [47]. Therefore, we performed a *LjPT4* expression time course [14], and detected an overlapping pattern with *LjNIP1* until 35 dpi. Interestingly, the correlation pattern was lost after this time, the aquaporin gene expression having remained high while *LjPT4* started to decrease. This indicated the moment at which arbuscules started to collapse in our experimental conditions [14]. In fact, mycorrhizal specific phosphate transporters have not been reported to be expressed in collapsed arbuscules [48].

**Figure 9:**
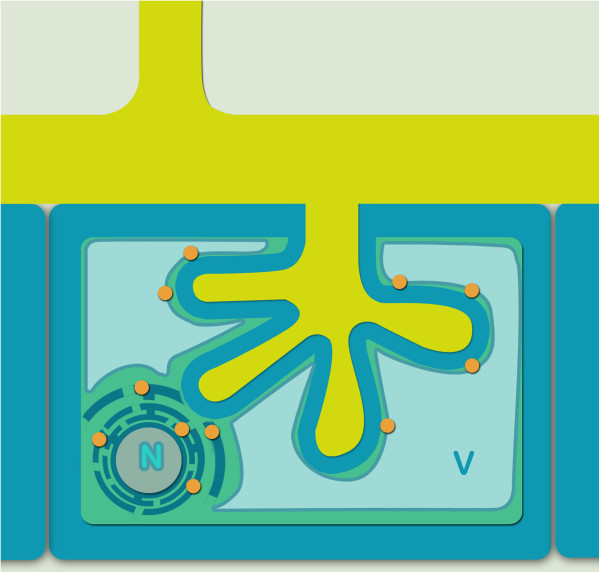
**Proposed model of the LjNIP1 role and localization.** The proposed LjNIP1 localization model (orange circles) within an arbusculated cortical cell. The scheme illustrates an intracellular fungal arbuscule, surrounded by the periarbuscular membrane. The vacuole (V) develops among the fungal branches and its tonoplast joins the periarbuscular membrane at some points. LjNIP1 is located in the endoplasmic reticulum surrounding the nucleus (N), in the inner membrane systems and in the tonoplast.

The transient expression of *LjNIP1*, linked to the green fluorescent protein in *Nicotiana* epidermal cells and in mycorrhizal *Lotus* roots, showed a localization in the endomembranes. The protein is surely associated with the nuclear membranes, a typical characteristic of the ER, but it also seemed to be dispersed in other endomembranes that could be the prosecutions of the ER (such as the Golgi apparatus) or the tonoplast, which proliferates in the colonized cells surrounding the arbuscule branches [49] and - as shown under the transmission electron microscope [50] – may adhere to the periarbuscular membrane at some points (Figure 9). A homologous aquaporin, *AtNIP2;1*, has been proposed to be involved in active cell elongation in the root elongating zone, where several kinds of organelles and the cell wall components are actively synthesized through the ER [51]: the same high physiological and morphological activity is surely necessary for the dramatic changes that take place in mycorrhizal cells.

In conclusion, we suggest that LjNIP1 is not exclusively localized in the periarbuscular membrane, like other proteins involved in nutrient uptake [52], but could be associated with other cell membranes, such as the tonoplast, the ER or ER-related membranes.

### LjNIP1 and LjXIP1 cannot transport ammonium or urea, but LjNIP1 can transport water

On the basis of the high sequence similarity of LjNIP1 and MtNIP1-2, which is capable of transferring ammonium, but not water [33], we verified whether LjNIP1 had the same characteristics, but obtained opposite results. In fact, while LjNIP1 was permeable to water, as shown by stopped-flow experiments, it was not capable of efficiently transferring ammonium or urea in yeast complementation assays. On the other hand, LjXIP1, like any Solanaceous protein from the same family, was not capable of transferring water [34] or other solutes.

These results have paved the way for various speculations, considering that *LjNIP1* resulted to be highly expressed from 21 dpi (days post inoculation) onwards. In fact, unlike previous descriptions, where the central vacuole was described as fragmented due to the presence of the fungus [53], it is now known, on the basis of electron microscopy and confocal observations, that the tonoplast follows the periarbuscular membrane, leading on one hand to an increase in the surface of the tonoplast during the arbuscule development and, on the other, to an intimate interaction between the two cell membranes [50,53-55] (Figure 9). We have speculated that LjNIP1 is involved in the maintenance of plant cell turgor via water passage within the inner membrane systems, or is directly involved in the passage of water from the fungus to the plant, a mechanism that is currently poorly understood [56]. Such a role is crucial when the arbuscular branches are active, as well as when they collapse and a higher quantity of water enters the vacuole to maintain the correct turgor. Since an important membrane biogenesis occurs in cortical cells before, during and after the colonization by the fungus [8], LjNIP1 could mediate the passage of water inside and outside the membrane of the different organelles. In conclusion we can assume that LjNIP1 functions as a water channel in the different membrane systems of the arbusculated cells.

## Conclusions

Taken as a whole, our findings reveal that two aquaporin-related genes belong to two distant families and respond differently to root symbioses, displaying a non-overlapping expression pattern. The potential role of *LjXIP1* remains rather obscure*,* although its exclusive response to AM fungal colonization, and not to N-fixing rhizobial bacteria, makes it a good candidate for further investigations. The arbuscule-specific *LjNIP1* has instead proved to be a novel AM symbiosis marker for the entire arbuscule life cycle. Due to the not-synchronous nature of AM colonization, *LjNIP1* seems to be a more versatile marker than *LjPT4*, as it covers a wider range of arbuscule development steps. The functional experiments have conclusively demonstrated that LjNIP1 does not transport ammonium, but can transfer water, unlike its *Medicago* homologous [33]. On the basis of these functional results, of membrane localization and of expression timing, it is tempting to assume that the protein is potentially involved, directly or indirectly, in cell turgor regulation, in facilitating colonized cell adaptation to osmotic stresses, and/or in the actual transfer of water from the fungus to the plant.

## Methods

### Plant materials, growth conditions and inoculation methods

*Gigaspora margarita* Becker and Hall (strain deposited in the Bank of European Glomales as BEG 34) was used as the fungal inoculum. The *Lotus japonicus* mycorrhization method (Regel) by K. Larsen (Gifu; wild type) has been described in detail by [14]. *Lotus* seedlings were grown in vermiculite for the nodulation experiment, and a B & D nutrient solution [57], containing 10 μM or 4 mM KNO_3_, respectively was used. A part of the N-starved plants was inoculated with *Mesorhizobium loti* strain NZP2235. Bacterial cells from a 2-d-old liquid culture (5 mL) were centrifuged, washed, and suspended in 50 mL of distilled water, and subsequently poured into the plant pots [58,59]. Twenty-eight days after fungal inoculation, samples were cut from the mycorrhizal roots after observation under a stereomicroscope to select roots with fungal mycelium. Root segments from three independent experiments were analyzed. At least three biological replicates were collected at different time points for the time course experiment.

### RNA isolation, cDNA synthesis and RT-PCR

The RNA isolation, cDNA synthesis, and quantitative RT-PCR (qRT-PCR) methods have already been described in detail [14]. Prior to qRT-PCR, gene-specific primers for *LjNIP1* and *LjXIP1* were tested on genomic DNA and cDNA for amplification purposes. Since RNA extracted from mycorrhizal roots contains plant and fungal RNAs, the specificity of the primer pair was also analyzed through PCR amplification of *Gi. margarita* genomic DNA. No amplification products were obtained on fungal DNA. The oligonucleotide sequences for the *LjNIP1* and *LjXIP1* were as follows: *LjNIP1* forward primer, 5^′^-ATTGGGTCTACATTACTGCT-3^′^; *LjNIP1* reverse primer, 5^′^-CTTGTCTGTGTATCTGAGGA- 3^′^. *LjXIP1* forward primer, 5^′^- TTGTGTCCATAACTGTGACC-3^′^; *LjXIP1* reverse primer, 5^′^- AATGTCCATTCCACACTGAG- 3^′^.

### 5′-RACE and 3′-RACE

5^′^-RACE and 3^′^-RACE were performed with the aforementioned total RNA extracted from the mycorrhized roots using a SMART RACE cDNA amplification kit (CLONTECH). The gene-specific primer sequences used are the same as those that were used in qRT-PCR. PCR was performed according to the CLONTECH protocol using the Advantage 2 PCR enzyme system and 35 cycles of 95°C for 30 s, 60°C for 30 s, 72°C for 2 min, and a final extension at 72°C for 10 min. The RACE products were subjected to electrophoresis, cloned in pCRII (TOPO cloning kit; Invitrogen), and analyzed through DNA sequencing.

### Plasmid constructs for yeast transformation

The coding region of *LjNIP1* (798 bp) was amplified from the aforementioned cDNA by means of PCR using the Advantage 2 PCR enzyme system (CLONTECH) and the following oligonucleotides, including XmaI/XhoI restriction sites which were then used for the subcloning: forward primer, 5^′^-TATCCCGGGATGGCTAACAATTCAGCTTC-3^′^; reverse primer, 5^′^-TATCTCGAGGGTAACTTTCTTCCATTTGC-3^′^. We used the following oligonucleotides for the isolation of the *LjXIP1* coding region (900 bp): forward primer, 5^′^- TATCCCGGGATGAATTCTTTTAACTCTCAGGTG-3^′^; reverse primer, 5^′^- TATCTCGAGTCAAGAAGCTTGAGGCAAC-3^′^. The PCR product was cloned in a pCRII vector (TOPO cloning kit; Invitrogen) and verified via full-length sequencing. The open reading frame of *LjNIP1* was subcloned into the yeast expression vector pDR199 for yeast (*Saccharomyces cerevisiae*) expression. The following AQPs were used as controls for the yeast expression experiment: AtAMT1;2 [36] and K10, a mutant form of AtPIP1;2 with a typical ar/R selectivity filter of AtNIP1-AtNIP4, which is able to transport any kind of compound [37].

### Expression in yeast

The plasmids containing the respective open reading frames were heat-shock transfected in the ura- AMT-defective yeast strain 31019b; ΔΔΔmep1;2;3 [60] and in the urea-; YNVW1; Δdur3; Δura3 [39]. The N-deficient growth medium was YNB without aminoacids, and ammonium sulphate (Difco), supplemented with 3% Glc and 3 mM NH_4_Cl as the only N source. No buffer was added. The yeast growth was not affected by the expression of the different constructs under non-selective conditions.

### Yeast protoplast preparation

Yeast protoplasts were prepared according to protocol [61], as previously described [62]. Cultures were grown in liquid SD-ura or SGal-ura on a rotary shaker for 18 h (250 rpm, 30°C). Cells from a 10-ml aliquot were spun down (500 × *g*, 5 min), resuspended in 3 ml of equilibration buffer (50 mM potassium phosphate at pH 7.2, containing 40 mM β-mercaptoethanol) and equilibrated on a rotary shaker at 30°C for 15 min. 6 ml of digestion buffer (50 mM potassium phosphate at pH 7.2, 40 mM β-mercaptoethanol, 2.4 M sorbitol, 50 mg/ml bovine serum albumin, 0.1–1 mg Zymolyase 20 T) was added. The mixture was vortexed and incubated on a rotary shaker for 45 min at 30°C. Protoplasts were harvested by means of centrifugation (1000 × *g*, 5 min), resuspended in an incubation buffer (1.8 M sorbitol, 50 mM NaCl, 5 mM CaCl_2_, 10 mM Tris brought to pH 8 with HCl) and kept on ice until use.

### Stopped-flow spectrometry

Volume changes in the yeast protoplasts, resulting from transmembrane water transport, were examined by means of 90° light scattering at 436 nm in a stopped-flow spectrophotometer (SFM 300, BioLogic). Yeast protoplasts were equilibrated in an incubation buffer (1.8 M sorbitol, 50 mM NaCl, 5 mM CaCl_2_ and 10 mM Tris/HCl, pH 8.0) and water transport was started by mixing the equilibrated protoplast suspension with an equal volume of test solution which had the same ionic composition but lower osmolarity (1.2 M sorbitol). This resulted in an outwardly directed osmotic gradient that induce water uptake and an increase in volume, which engenders a decrease in the intensity of the scattered light.

### Water permeability measurements

The water permeability of the intact yeast protoplasts was measured by means of stopped flow spectrophotometry [61]. The protoplasts were exposed to a 300 mosmol outwardly directed osmotic gradient in order to induce swelling. Changes in volume were measured considering the decrease scattered light intensity in a stopped flow spectrophotometer (SFM-300, Bio-Logic SAS, Claix, France). Quantification of water conductivity was achieved by fitting a single exponential function to the initial 100 ms of the swelling kinetics using Biokine (Bio-Logic SAS) software. The curves were drawn after at least five independent experiments of three independently transformed clones, with an average of 20 measurements each (n ≥ 100).

### Laser microdissection

Mycorrhizal and nonmycorrhizal root segments were fixed in freshly prepared absolute ethanol:glacial acetic acid (3:1) at 4°C overnight for paraffin embedding [63]. A Leica AS laser microdissection system (Leica Microsystems) was used to isolate cells from the prepared tissue sections, as described in previous works [14,63]. After collection, the RNA extraction buffer from a PicoPure kit (Arcturus Engineering) was added and samples were incubated at 42°C for 30 min, centrifuged at 800 g for 2 min, and stored at −80°C. Then, for the following RNA extraction steps, about 1,500 cells were pooled, for each cell-type population, in single tubes, in a final volume of 50 μL.

### RNA extraction, RT-PCR and qRT-PCR on microdissected samples

The RNA extractions were performed following a slightly modified PicoPure kit protocol (Arcturus Engineering), as described in other works [63]. RNA quantification was obtained using the NanoDrop 1000 spectrophotometer. A One-Step RT-PCR kit (Qiagen) was used for the RT-PCR experiments, that were conducted on the RNA extracted from several samples. Reactions were carried out as described in detail in [14]. Amplification reactions were carried out with specific primers for *LjNIP1, LjXIP1* and the housekeeping gene *LjEF1a*[14]; with annealing temperatures of 65°C and 60°C, respectively. The RT-PCR experiments were conducted on at least three independent biological and technical replicates. A One-Step qRT-PCR kit (Biorad) was used for the qRT-PCR experiments on microdissected cells. Reactions were carried out on two biological and two technical replicates according to the manual’s instructions and using the same primers and annealing temperatures mentioned above. In each well, a final volume of 20 μL was composed of 12,5 μL of 2X Syber Green, 0,5 μL of primer 10 μM (forward and reverse), 0,5 μL of Reverse Transcriptase enzyme and RNase free water.

### *Nicotiana* leaf epidermal cell transformation and confocal analysis

pK7FWG2,0 for C-terminal GFP fusion and expression under the 35S promoter of CaMV promoter was introduced into *Agrobacterium tumefaciens* GV3101, and *Nicotiana tabacum* leaves were then agroinfiltrated according to the protocol published by Batoko and coworkers [64]. Confocal microscopy analyses were performed using a Nikon PCM2000 (BioRad) laser scanning confocal imaging system. The excitation for GFP detection was at 488 nm and the detection was between 515 and 530 nm. The excitation for the chlorophyll detection was at 488 nm and the detection was over 570 nm. The images acquired from the confocal microscope were processed using Corel Photo-Paint software (Corel Corporation, Dallas, TX, USA).

### Subcellular localization in mycorrhizal roots

The *LjNIP1* coding region was amplified from cDNA using the following primers: *LjNIP*-attB-forward/reverse. The amplified fragment was inserted into pDONR221 (Invitrogen) and then recombined using the Gateway system (Invitrogen), in the binary vector pK7WGF2,0 for subcellular localization [65]. The red fluorescent marker DsRED was inserted under the control of the constitutive *Arabidopsis* Ubiquitin10 promoter (P*Ubq10*) [66]. An *LjNIP* promoter fragment of 1200 bp was PCR-amplified from genomic DNA using primers p*LjNIP*-forward/reverse containing SacI and SpeI site, respectively. The promoter fragment was used to replace the CaMV 35S promoter in the pK7WGF2,0 that contained the *LjNIP1* coding region.

The *pLjNIP1*-*eGFP*-*LjNIP1* construct was used to stably transform *L. japonicus* roots via *Agrobacterium rhizogenes*. Root segments showing DsRED fluorescence, colonized by *Gi. margarita*, were excised and included in 8% agarose. The resulting agarose block was cut into thin slices (200 μm) using a vibratome and the slices were placed on a slide. Each section was observed using a Leica TCS-SP2 confocal microscope fitted with a long-distance 40x water-immersion objective (HCX Apo 0.80). GFP was excited with a blue argon ion laser (488 nm) and the emitted fluorescence was collected between 500 and 545 nm. DsRED was excited at 488 nm and imaged at 600–700 nm. The greenish autofluorescence of collapsed hyphae was partially captured by the GFP emission window under these imaging conditions. Data was collected from a minimum of 10 independently transformed root lines.

## Competing interests

The authors declare that they have no competing interests.

## Authors’ contributions

MaG carried out the experiments and drafted the manuscript. RB contributed to the LMD experiments. VV helped with the plasmid constructions, *Lotus* transformation and time-course analyses. MiG participated in the design of the work and performed the first qRT-PCR. DS and UL performed the functional complementation assays and the stopped-flow experiments. AC performed the tobacco leaf cell transformation. PB coordinated the project and wrote the manuscript. All authors read and approved the final manuscript.

## Supplementary Material

Additional file 1 **Aminoacidic sequence alignment of LjNIP1 and LjXIP1.** Sequence alignments of LjNIP1 and LjXIP1 with the closest protein found with Blast. The amino acid sequences were compared using CLUSTALW software. The two ‘NPA motifs’ are coloured in light grey. The four residues (R1–R4) forming the selectivity filter are shaded in dark grey. Click here for file

Additional file 2 **Prediction of transmembrane helices in LjNIP1 and LjXIP1.** The TMrpres2D web tool (http://bioinformatics.biol.uoa.gr/TMRPres2D/) allows us to confirm the putative 6 transmembrane domains, which are typical of the aquaporin class. Click here for file
