# Supplementary material for: Two putative-aquaporin genes are differentially expressed during arbuscular mycorrhizal symbiosis in Lotus japonicus
Source: BMC Plant Biol. 2012 Oct 9;12:186. doi: 10.1186/1471-2229-12-186 (PMC3533510; doi:10.1186/1471-2229-12-186)
Supplement: Additional file 1 — Aminoacidic sequence alignment of LjNIP1 and LjXIP1. Sequence alignments of LjNIP1 and LjXIP1 with the closest protein found with Blast. The amino acid sequences were compared using CLUSTALW software. The two ‘NPA motifs’ are coloured in light grey. The four residues (R1–R4) forming the selectivity filter are shaded in dark grey. [file 1471-2229-12-186-S1.pdf]

```
LjNIP1     MAN-NSASFETHDVVLSVNKDASKTIESSDTYTSVFFVQKLVAEFVGTFFLIFTGCASIVVNKNNDNVVTLPGIALVWGLVLMVLIYSVG 89
MtNIP1.2   MANDNSARIETHEVVLDTNKDSSDTCKGSGSFVSVPFLQKLIAEMVGTYFLIFAGCASIVVNKDNDNVVTLPGIAIVWGLTLLVLIYSLG 90
                                                                                        R1

LjNIP1     HISGAHFNPAVTFAFATTKRFPWIQVAPYIASQLLGAVLASGILKMLFSGASQLLGAVLASGILKMLFSGTHDQFSGTIPSGTNLQAFVI 179
MtNIP1.2   HISGAHFNPAVTIAFATTRRFPLLQVPAYISAQLLGATLASGTLKLIFSGSAQLLGATLASGTLKLIFSGAHDHFSGTLPSGSNLQAFVL 180

LjNIP1     EFITTFLLMFVISAVATDNRAIGEMAGIAIGSTLLLNILISGPITGASMNPARTLGPAIFHSKYRAIVVYFVSTIFGAVAGAWVFNILRY 269
MtNIP1.2   EFITTFYLMFTISGVATDTRAIGELAGIAIGSTLLLNVMIAGPVTGASMNPVRTLGPAFVHNEYRGIWIYLLSPILGAIAGAWVYNTVRY 270
                                                R2       R3     R4

LjNIP1     TDKPLHEITKGSSILK---------- 285
MtNIP1.2   TNKPLREITQSASFLKEAGRGGHQIV 296


LjXIP1     MNSFNSQVADEQLSRYVQTSNERKNAS------------RGK---FLAS---IGVHEIFRSETWKAAMTELAATASLVFTLTTSIIACLD 42
SlXIP1     MASNSNVVFGDEESQISGGTNRVQPCSSTPRKINTIDDDEGKKHNFLSLSQRLGVADFFCLDVWRASMGELLGSAVLVFMLDTIVISTFE 60

LjXIP1     SHETDPKLLVPFVVFIIAFLFLIVTVPLSGGHMSPVFTIIAALKGVVTLTRALIYMFAQCVGSIIGFFVLKCVMDPKLVHTYSLGGCTI- 161
SlXIP1     SETKMPNLIMSILIAVVITILLLAVVPVSGGHINPVISFSAALVGIISMSRAIIYIVAQCVGAILGALALRAVVSSIEDTFSLGGCTVT 180
                     R1

LjXIP1     ----GGNG-VNSAITEYDALLLEISCTFLVLFLGVTLAFDKKRAKELGLPMVCMVVAGAMALAVFVSITVTGRPGYAGVGLNPARCLGPA 246
SlXIP1     IIAPGPNGPVIVGLETAQALWLEIFCTFVFLFASIWMAYDHRQAKALGHVTVLSIVGLVLGLLVFISTTVTAKKGYGGAGINPARCLGPA 270
                                                                                   R2       R3     R4

LjXIP1     LLQGGSVWNGHWVFWVGSFLACIIYYVVSVNLPKE----GSGIGDGEFDVVKLPQAS 299
SlXIP1     IIRGGHLWDGHWIFWVGPTIGCVAFYVYTKIIPTKHFLAEYGFKHDFVGVVKALSNV 327
```
