# Supplementary figures and images for: Two putative-aquaporin genes are differentially expressed during arbuscular mycorrhizal symbiosis in Lotus japonicus
Source: BMC Plant Biol. 2012 Oct 9;12:186. doi: 10.1186/1471-2229-12-186 (PMC3533510; doi:10.1186/1471-2229-12-186)

CYTOPLASMIC

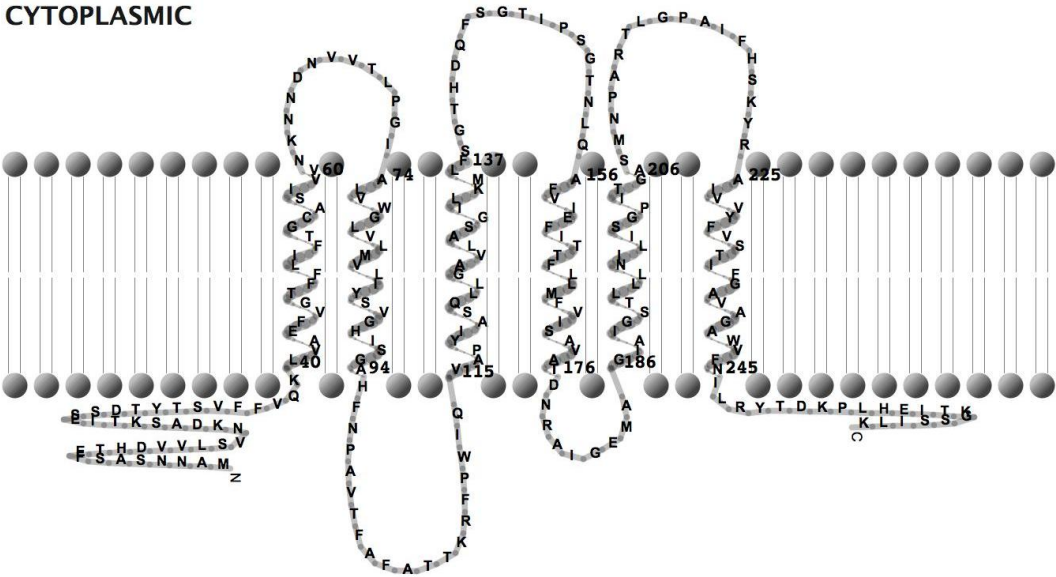

LjNIP1

EXTRACELLULAR

CYTOPLASMIC

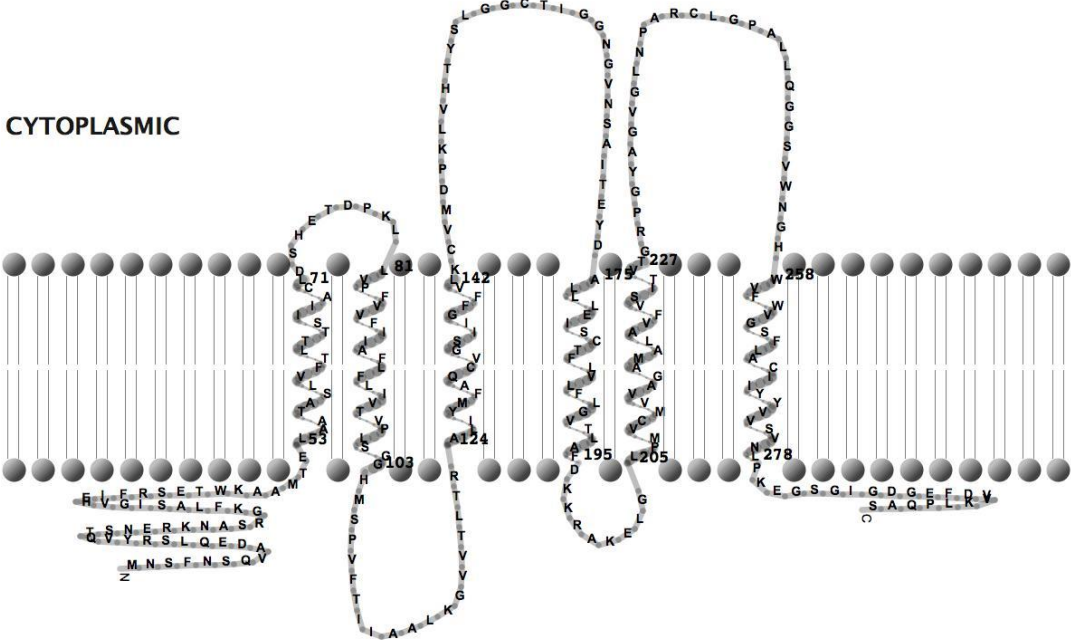

LjXIP1

EXTRACELLULAR

Supplement: Additional file 2 — Prediction of transmembrane helices in LjNIP1 and LjXIP1. The TMrpres2D web tool (http://bioinformatics.biol.uoa.gr/TMRPres2D/) allows us to confirm the putative 6 transmembrane domains, which are typical of the aquaporin class. [file 1471-2229-12-186-S2.pdf]
